# An ode to multidisciplinarity: the ‘Bacteria Orchestrate Life’ international meeting

**DOI:** 10.1242/bio.060304

**Published:** 2024-03-01

**Authors:** Mohamed Jemaà

**Affiliations:** ^1^ Human Genetics Laboratory, Faculty of Medicine of Tunis, Tunis El Manar University, Tunis 1006, Tunisia; ^2^ Department of Biology, Faculty of Science of Tunis, Tunis El Manar University, Tunis 2092, Tunisia

**Keywords:** Multidisciplinarity, Bacteria, Open science, Workshop, Conference

## Abstract

If scientists stick to their research expertise without collaborating with other experts in different fields, it could stall the progress of their work in a world where interdisciplinary thinking and working should be second nature. Biologists are at the forefront of this trend. That is why a consortium formed by the faculty of sciences of Tunis El Manar University, Tunisia, the GetGenome Foundation and Learn and Win, decided to organise an international conference on bacteria, a perfect field for multidisciplinarity. For 3 days, from 14 to 16 December 2023, more than 200 interdisciplinary researchers and students of life sciences and more than 20 international speakers and trainers met at the faculty of sciences in Tunis, to discuss microbiology and bacteria from different horizons, from the most fundamental to the most imaginative, with a strong focus on technologies and discoveries. This Meeting Review describes the scientific event and highlights the main results of both the conferences and the practical sessions.

## Teaser

**Summary:** From 14–16 December 2023, more than 200 interdisciplinary researchers, students of life sciences and more than 20 international speakers and trainers met at the Faculty of Sciences in Tunis, Tunisia, to discuss microbiology and bacteria from different horizons. This Meeting Review describes the event and highlights the key papers and the practical sessions.

## Multidisciplinarity in science

I once had a discussion about multilingualism with a polyglot who worked in the laboratory where I did my PhD. What I took away from that discussion was that one of the very important things about speaking and understanding several languages is that you learn that the meaning of a word is concept-dependent, that what is true in one context is false in another context and vice versa, so you learn to relativise how you perceive reality and how you see it, and it makes you more flexible in the way you deal with problems.

In science, the same notion applies. Multidisciplinarity refers to the integration of knowledge, methods and perspectives from different disciplines to address a complex problem in a flexible way. It involves collaboration and interaction between experts from different scientific fields, each bringing their unique insights and approaches to the common research effort (Alvargonzález, 2011).

In terms of opportunities and excitement, multidisciplinarity has a lot to offer to early career researchers, especially in low-income countries with limited research and technology budgets. Indeed, in addition to the high-quality research that results from such collaborations, it is also effective time and resource management: collaborative research certainly costs less than a single laboratory marathon (Chambliss et al., 2019; Abramo et al., 2018; King et al., 2013).

## Why a multidisciplinary conference on bacteria?

Microbes and bacteria are literally everywhere, in the soil (Timofeeva et al., 2023), in water (Cabral, 2010), in animals and humans (Dekaboruah et al., 2020), in plants (Buttimer et al., 2017), in extreme environments (Rampelotto, 2013), and even in space stations (Koehle et al., 2023). Bacteria are also used as tools in many contexts, especially in health and disease (Tanniche and Behkam, 2023). They are everything, everywhere, all at once! And this definitely requires an interdisciplinary research approach to study the field, sometimes involving even historians and anthropologists, in addition to biologists (Amato et al., 2019). As reported in several recent projects, microbial research requires original approaches, for example, the study of the complexity of the microbiome in the context of public health requires translational and clinical investigation, but it also involves diagnostic and treatment modelling, which require the involvement of physiologists and clinicians, as well as accountants and statisticians to evaluate cost-effectiveness models (Mylonakis, 2023; Berg et al., 2020; Booth et al., 2023). Basic bacterial strain research, for example in health and disease or environmental studies, is challenging traditional standards and now involves a combination of phylogenetics, bioinformatics and biochemistry (Ceniceros et al., 2021). Microbes also means biofilms, and this field needs a biophysical vision in addition to microbiologists, and in future it will need the help of economists to grasp the financial implications of biofilm research for different sectors, including industry (Camara et al., 2022).

Bacteriology therefore is the perfect subject to bring together multidisciplinary scientists to talk about how bacteria orchestrate life. The best audience for the conference was young students and researchers. To organise such an exciting scientific event, I chose the Faculty of Sciences of Tunis (FST) (https://fst.rnu.tn/en) as the main venue. The FST is a prominent educational institution in the capital city of Tunis and is considered one of the largest and most prestigious institutions in Tunisia.

## What were the key themes of the meeting?

The conference kicked off on Thursday 14 December 2023 with James Canham from the Sainsbury Laboratory, Norwich, UK giving a presentation entitled ‘GetGenome: Genomics For All!’ to introduce the GetGenome Foundation and its mission to empower scientists by providing equitable access to genomics technology and genomics-related training and education. Barrie Wilkinson from the John Innes Centre, Department of Molecular Microbiology, Norwich Research Park, UK, took the lead with a talk entitled ‘Antibiotics: past, present and future’, and enlightened us on the biosynthesis of microbial natural products. Then, Alif Chebbi, from the Faculty of Science, Roma Tre University, Italy, gave a talk entitled ‘Cave microbiomes in the era of omics: current and future challenges’ and shared his experience of caving and sequencing these types of inaccessible microbes. Yogesh Singh from the Medical Genetics and Applied Genomics NGS Competence Centre in Tübingen, Germany, concluded the first day session with a presentation entitled ‘Gut–brain axis: gut microbiome and neurodegenerative diseases (NDDs)’ and explored the links between the brain and gut microbiota.

Day 2 began with a presentation by Naouel Klibi from the Faculty of Sciences, Tunis, ‘Why do bacteria need plasmid?’, followed by Madhuri S Salker from the Research Institute of Women's Health, Eberhard Karls University, Tübingen, Germany, on ‘The use of plasmids to understand female fertility’. Joseph Sallmen from the John Innes Centre, Department of Molecular Microbiology, Norwich Research Park, UK, focused on more fundamental research into bacterial growth and division and gave a talk entitled ‘CIS-tem of a Down: a mechanism for how a multicellular bacterium responds to stress and development’. Nissem Abdeljelil from the Research Institute for Biosciences, University of Mons, Belgium took us to the International Space Station to search for microbial biofilms with a talk entitled ‘Challenging the biofilms: space, radiations and chemical stress’ and Skander Hathroubi from SPARTHA Medical, University of Strasbourg, France continued with a talk entitled ‘Bacterial biofilms, lessons from nature’.

Day 3 was also a high-quality conference, starting with a focus on water pollution, and Hana Trigui from Polytechnique Montréal, Université d'Ingénierie, Canada, with a lecture entitled ‘Microbiology of surface and ground water’. Afterwards, still in the water, Yassine Ramzi Sghaier of the TunSea Association, Tunisia, took the lead and demonstrated the interaction between marine plants and bacteria with a presentation entitled ‘Bacterial symbiosis in sea grass’. We then moved on to the botanical side, and Hédia Bourguiba from the Faculty of Sciences in Tunis gave a presentation entitled ‘Does the apricot biome benefit plant health?’ Mohamed Jemaà from the Faculty of Sciences of Tunis closed the conferences with a curious talk mixing biology and archaeology, showing how scientists defined the true passage of Hannibal through the Alps during the Punic War, talk entitled ‘Hannibal *ad portas*: chemical biomarkers and microbial signatures’.

Each day there were lots of questions and interactions from participants making connections between what they were learning at the conference and what they were working on themselves. The scientific melting pot we had was really amazing.

**Fig. 1.**
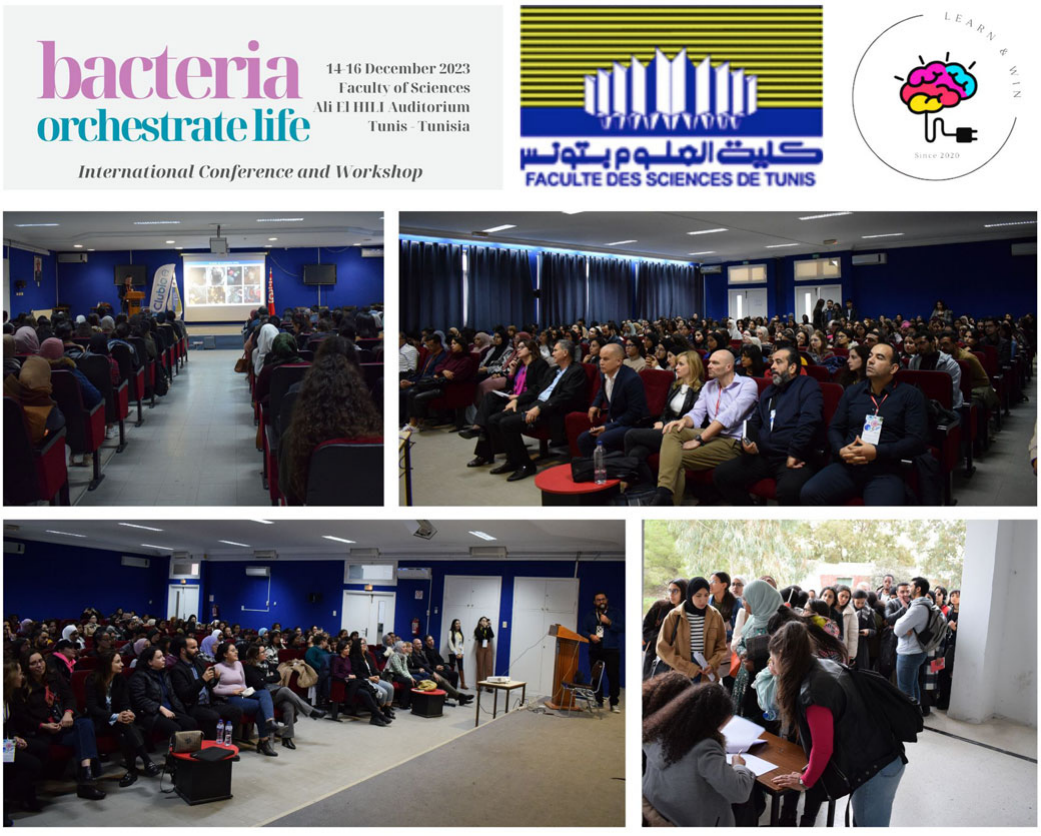
Daily attendance overview for conferences.

The 3-day seminar was in open access without any fee, but the pre-registration was mandatory. We had almost 400 pre-registrations and about 150 participants in the amphitheatre each day (Fig. 1). Recordings of the conferences are available on YouTube (https://www.youtube.com/@learnwin2050/videos).

## Workshop and practical sessions

In addition to the conference, we also organised practical sessions on bacterial DNA and plasmid extraction for selected candidates. We received a large number of applications and, due to the limited number of places, we selected only Masters students to enrich their training and planned a second edition, April 2024, for PhD students and postdocs to allow them to master these biomolecular techniques.

**Fig. 2.**
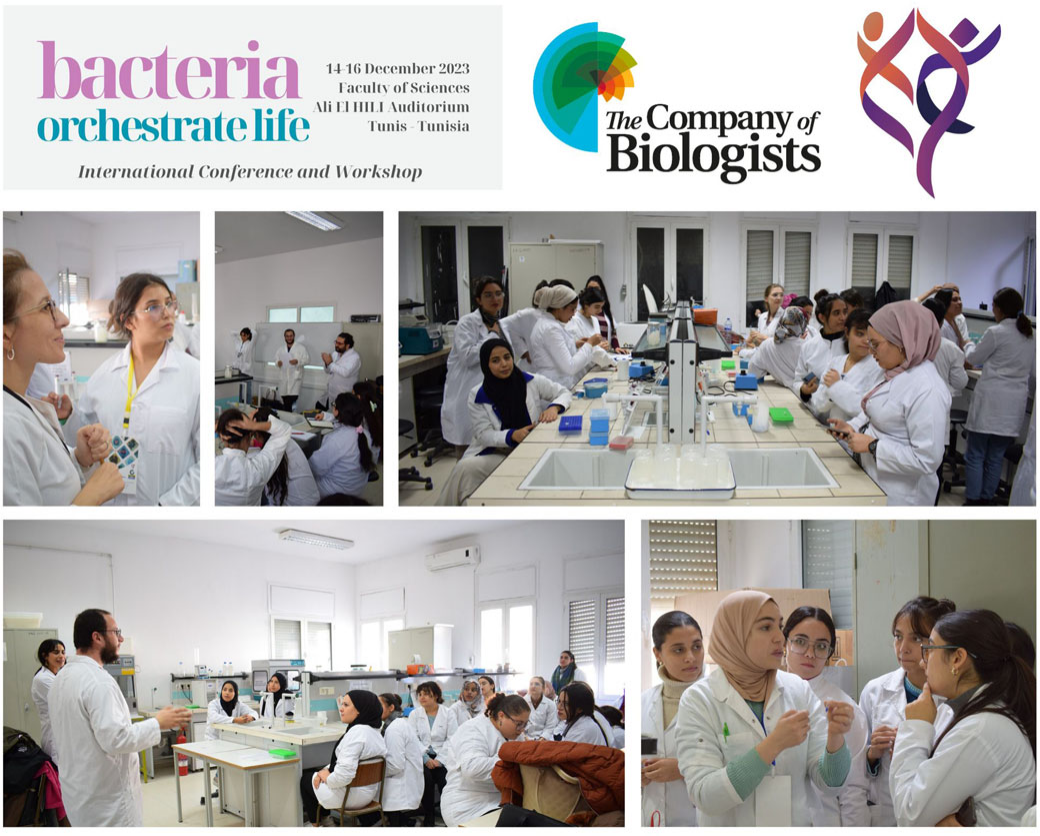
An overview of the practical sessions.

The practical sessions were structured as follows, first we introduced the participants to the concept of bacterial plasmid and genomic DNA, then we started two protocols for plasmid extraction, the first was the use of a commercial KIT and the second was the use of a home-made alkaline lysis method to lyse bacterial cells and separate genomic DNA from plasmid DNA. Quantification and visualisation of the final DNA and quality control were also performed. The main objective was to evaluate the low budget method and its suitability for DNA sequencing (Fig. 2).

## Take-home message

When experts from different fields work together on a common topic within the boundaries of their own discipline, this is called a multidisciplinary approach. This is what we tried to facilitate with our conference ‘Bacteria Orchestrate Life’. Conference take home messages were: (1) global approaches are key: participants emphasised the need for holistic and comprehensive approaches to problem solving. Solutions are more robust when they take into account multiple perspectives and factors. (2) Collaboration is essential: there is no more effective brainstorming than bringing together experts from different fields to tackle complex challenges. (3) Flexibility: researchers need to be open to different methodologies, terminologies and ways of thinking, even if they are unfamiliar. (4) Synergy: combined efforts from diverse disciplines create *de facto* synergies that lead to innovative solutions and new perspectives. (5) Continuous learning: researchers need to be constantly learning and adapting, and be open to new ideas and methods from other fields. (6) Communication is key: understanding the challenges of interdisciplinary communication and emphasising the importance of effective communication between experts from different fields encourages the development of a common language, which will facilitate collaboration.

Participants encourage young researchers to embrace the philosophy of multidisciplinarity, to attract effective collaborations, and believe that collaboration will lead to improved research and education.
